# Rapid Post-Earthquake Structural Damage Assessment Using Convolutional Neural Networks and Transfer Learning

**DOI:** 10.3390/s22093471

**Published:** 2022-05-03

**Authors:** Peter Damilola Ogunjinmi, Sung-Sik Park, Bubryur Kim, Dong-Eun Lee

**Affiliations:** 1 School of Architecture, Civil, Energy, and Environment Engineering, Kyungpook National University, 80 Daehakro, Bukgu, Daegu 41566, Korea; peterogunjinmi@knu.ac.kr; 2 Department of Civil Engineering, Kyungpook National University, 80 Daehakro, Bukgu, Daegu 41566, Korea; sungpark@knu.ac.kr; 3 Department of Robot and Smart System Engineering, Kyungpook National University, 80 Daehakro, Bukgu, Daegu 41566, Korea

**Keywords:** transfer learning, convolutional neural network, earthquake, image classification, damage detection

## Abstract

The adoption of artificial intelligence in post-earthquake inspections and reconnaissance has received considerable attention in recent years, owing to its exponential increase in computation capabilities and inherent potential in addressing disadvantages associated with manual inspections. Herein, we present the effectiveness of automated deep learning in enhancing the assessment of damage caused by the 2017 Pohang earthquake. Six classical pre-trained convolutional neural network (CNN) models are implemented through transfer learning (TL) on a small dataset, comprising 1780 manually labeled images of structural damage. Feature extraction and fine-tuning TL methods are trained on the image datasets. The performances of various CNN models are compared on a testing image dataset. Results confirm that the MobileNet fine-tuned model offers the best performance. Therefore, the model is further developed as a web-based application for classifying earthquake damage. The severity of damage is quantified by assigning damage assessment values, derived using the CNN model and gradient-weighted class activation mapping. The web-based application can effectively and automatically classify structural damage resulting from earthquakes, rendering it suitable for decision making, such as in resource allocation, policy development, and emergency response.

## 1. Introduction

Classification of the magnitude of damage to buildings and infrastructure attributed to seismic events is essential for enhancing post-earthquake reconnaissance and ensuring safe and effective recovery efforts. Conventionally, property damage attributed to earthquakes is documented manually using labor-intensive methods [1,2,3,4,5]. Manual damage inspections may be time consuming and involve arbitrary judgment by a novice inspector who may not be adequately trained. These disadvantages can be addressed by performing fully automated inspections using computer-vision technologies [6]. The automated deep learning (DL) method may be critical for enabling the rapid real-time detection and classification of structural damage (SD) attributed to earthquakes.

DL algorithms for image classification may be applicable for assessing SDs [6,7,8,9,10,11]. Gao and Mosalam [6] created an image database known as “Structural ImageNet,” which implements a visual geometry group (VGG) convolutional neural network (CNN) model through transfer learning (TL) to classify SD caused by earthquakes. They curated the Pacific Earthquake Engineering Research (PEER) Hub ImageNet [12] dataset, which serves as a benchmark for similar computer-vision-based classification and detection tasks [13]. Nahata et al. [7] employed the VGG16 TL model to classify post-earthquake building damage into four categories. After training the model with more than 1,200 images, they obtained training and validation accuracies of 97.85% and 89.38%, respectively. In addition, DL methods have been exploited for damage-detection tasks, in which bounding boxes are used to identify and localize SD [14,15]. 

Decision makers can allocate the appropriate resources to retrofit, repair, and recover facilities by locating and quantifying SDs. A numerical scale that quantifies the magnitude of SD to facilitate such efforts has been established. Li et al. [16] identified a mismatch in the damage detected using conventional approaches. They proposed a novel approach to quantify the severity of SD, using a smooth image heat map based on gradient-weighted class activation mapping (Grad-CAM). In fact, this approach has been employed in several applications, such as post-disaster damage assessments [10] and steel frame damage investigations [17], and demonstrated performances superior to or comparable with other state-of-the-art methods, while requiring low computation time. We employed the approach to quantify and locate SD caused by the 2017 Pohang earthquake using CNN based on TL strategies. Through TL, CNN models can learn complex patterns from data without needing a large amount of training data. Additionally, they can generalize well to new datasets, which is important when dealing with SD that may vary in appearance from one instance to another. Therefore, the performance of Feature Extraction (FE) and Fine Tuning (FT) TL methods on SD image datasets were compared, in order to explore the possibility of applying the knowledge from a pre-trained model (source domain) to another (target domain), by tuning some of the model parameters. Finally, the optimal CNN model used to implement the approach was deployed on an interactive webpage that automatically classifies SD caused by earthquakes. Invisible damage, which is beyond the scope of this study, is typically examined via anomaly detection in structural members using specialized sensors and signal-processing techniques. However, in the abovementioned study, damage was considered visible to either the human eye or computer vision. This novel approach can facilitate rapid responses following an earthquake. Researchers have successfully identified SD characteristics using classification [6,7], bounding box detection [8], and segmentation techniques [9]. However, most of those methods do not involve a tool with a post-disaster assessment framework that is accessible to the structural engineering community.

Moreover, only a few studies have considered the deployment of post-earthquake damage classification, rendering it less useful for industrial applications and field validation. By contrast, both object localization with Grad-CAM and model deployment for practical applications are considered in the current study. This novel approach is relevant to researchers and practitioners as it fills the research gap by providing an interactive tool for SD assessment.

**Figure 1:**
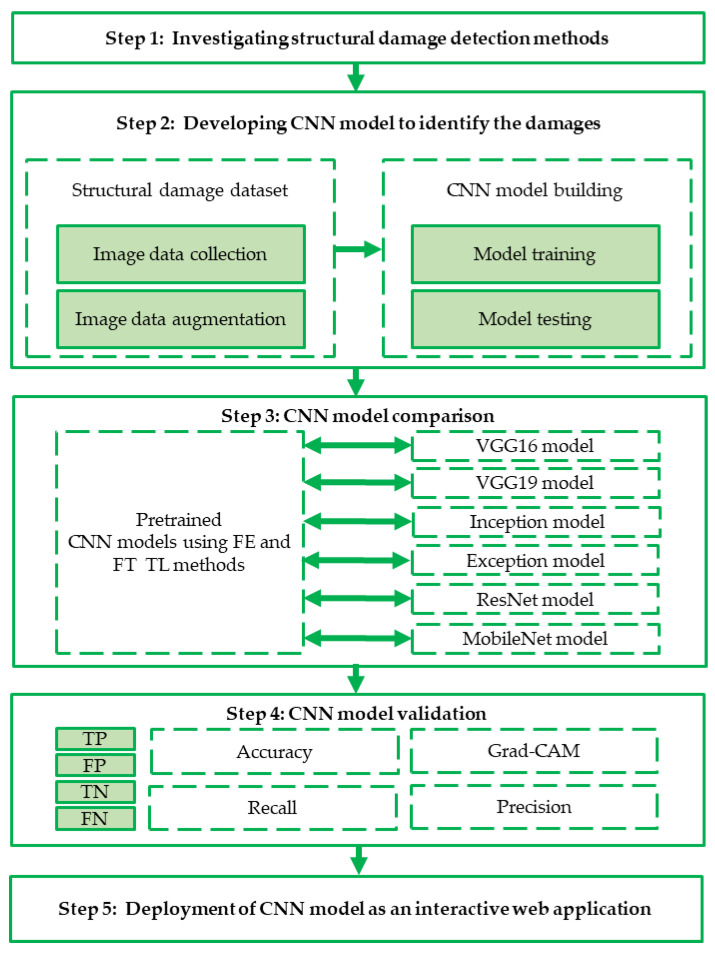
Workflow of the research method used in current study.

The remainder of this paper is organized as follows: Section 2 presents a brief overview of related studies. Section 3 describes the data acquisition process and methodology, and Section 4 discusses the results of the CNN model training, damage localization, and quantification. Section 5 presents an interactive webpage for damage classification, and Section 6 presents the conclusions and future research directions. The workflow of the research method is shown in Figure 1.

## 2. Related Studies

This section provides a brief overview of existing studies related to the current study. A comprehensive review of the applications of computer-vision-based civil infrastructure inspection has recently been presented [18]. Pan and Yang [8] implemented an object detection algorithm to quantify damage to structural elements and the associated repair costs. Their proposed algorithm achieved average precisions of 98.2% and 84.5% on the training and testing image datasets, respectively. For automated post-earthquake inspection, Hoskere et al. [9] proposed a multiscale deep CNN, incorporating ResNet23 and VGG19 as damage classifiers and damage segmenters, which achieved accuracies of 88.8% and 71.4%, respectively. Liang [11] investigated an image-based approach for inspecting bridges by considering system, component, and local damage level detection. The proposed DL network comprises a pre-trained VGG-16 CNN for system-level failure classification, a faster region-based CNN for component-level bridge column detection, and a fully convolutional network for damage segmentation. Bayesian optimization enhanced the model performance and afforded an accuracy exceeding 90% for all the three-level tasks considered.

Some disadvantages in the existing multiclass damage assessment approach mentioned earlier include dataset class imbalance, which results in overfitting, lack of scalability and flexibility of the CNN architecture for solving various challenges, noisy training data, and a complex CNN architecture [19]. Therefore, recent applications of CNN-based models for SD assessments focus more on quality data preparation, the algorithmic optimization of the CNN model architecture, and damage quantification. Techniques typically adopted for quality image data preparation include image enhancement approaches, such as gray-level thresholding, histogram equalization, and adaptive histogram equalization [20]. Moreover, the algorithmic optimization of hyperparameters enhances the accuracy of CNN-based models and reduces the computational power used for execution [21]. Recently, Kim et al. [22] developed an optimized LeNet (OLeNet) model by tuning a shallow LeNet-5 CNN architecture for concrete surface crack detection. Consequently, OLeNet achieved an optimum validation accuracy of 99.8% at 19 epochs within 220 s of model training. Meanwhile, pre-trained deep CNN architectures, including ResNet, VGG16, and Inception, required at least 45 epochs to achieve the same validation accuracy within 524 s.

## 3. Methodology

### 3.1. Data Acquisition, Division, and Preprocessing

A total of 2750 images were acquired from field investigations [1,2,3,4,5] for different earthquakes. This study focuses on the Pohang earthquake. However, data obtained from other earthquakes were used to build a robust model to increase generalizability. A summary of the image datasets is presented in Table 1. Light damage indicates hairline cracks in structural elements, whereas moderate damage indicates wider cracks and spalling in concrete. By contrast, severe damage represents elemental collapse or structural failure [23]. 

**Figure 2:**
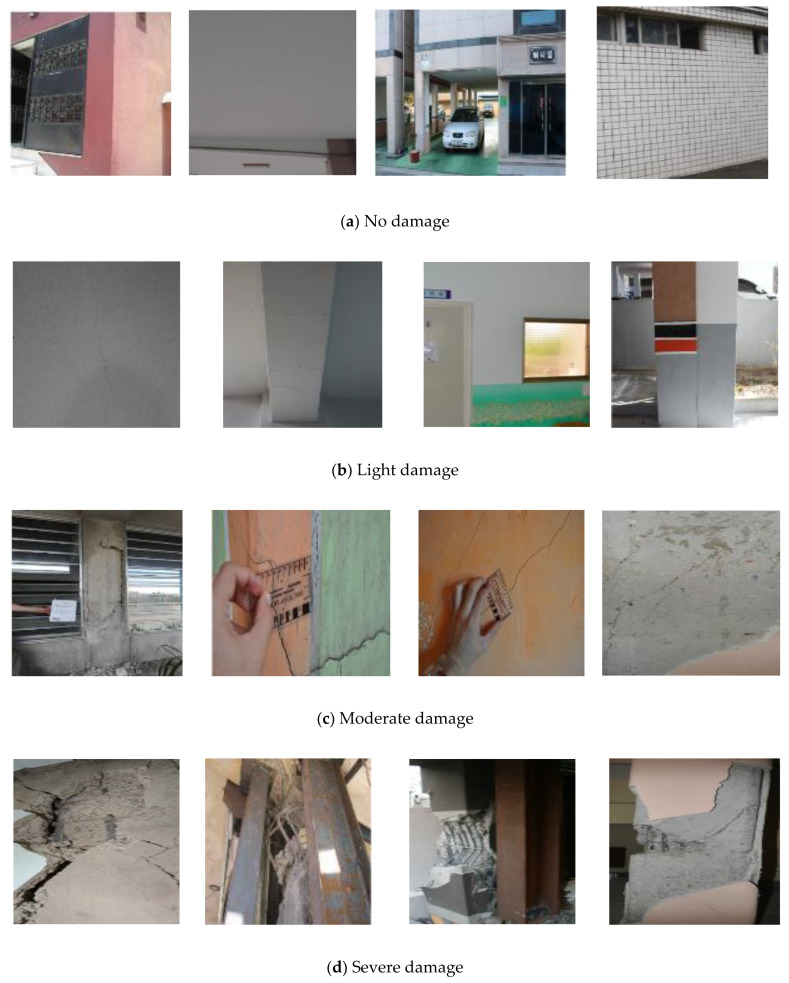
Samples used for training images for each damage class: (**a**) no, (**b**) light, (**c**) moderate, and (**d**) severe damage.

The methodology involves a supervised learning image classification problem. Therefore, the labeled image dataset was split into two to train and evaluate the model’s performance after each epoch. The ratio of the training and validation sets was empirically set at 4:1. In addition, the validation datasets were used to test the training performance of the models after each epoch. A total of 1780 images were selected from the database, of which 1600 were used for training and validation (Table 2). To address the data imbalance during model training, each damage class was penalized by assigning class weights of 1.0, 1.5, 1.5 and 2.4 to the severe, light, moderate, and no damage classes, respectively. A total of 180 images were obtained exclusively from the damage database of the Pohang earthquake and these were used to evaluate the generalizability of the trained model. Figure 2 shows a sample of 1600 images selected to train the CNN model.

### 3.2. TL Using Pre-Trained CNN Models

Six pre-trained classical CNN setups were implemented via TL. TL is an efficient approach used for training a small dataset, whereby a neural network pre-trained on a large dataset in the source domain is applied to the target domain. The underlying hypothesis of TL is that common features learned from a sufficiently large dataset are transferred to different datasets [24]. For practical applications, two strategies are used while conducting TL in deep CNNs: feature extraction (FE) and FT. We used FE and FT TL methods to train the models on the datasets. In the FE method, the fully connected layers are removed from a network that has been pre-trained on the ImageNet dataset, while maintaining the convolutional base as a feature extractor. The pre-trained network serves as an arbitrary feature extractor that performs convolutional operations once on the input image during forward propagation, stops at the pre-specified layer, and uses the outputs of that layer as bottleneck features. In summary, the pre-trained CNN models serve as the backbone for FE, in which all the parameters in the convolution layers are frozen, whereas the fully connected layers are updated during backpropagation [25].

**Figure 3:**
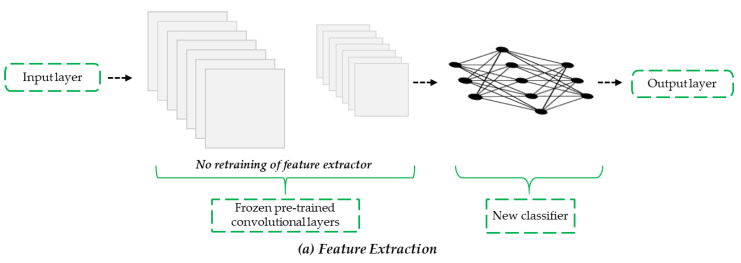

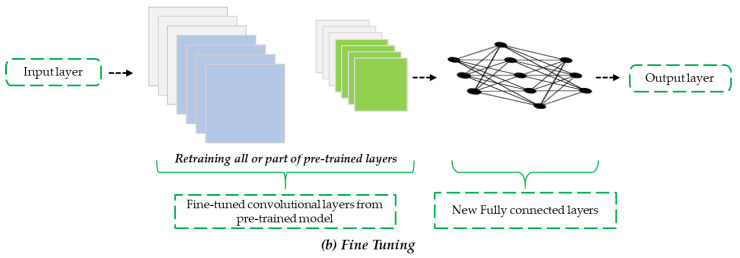
Procedure for TL using (**a**) FE and (**b**) FT.

However, the FT method requires the unfreezing and retraining of the pre-trained convolutional base through backpropagation. During retraining, the convolutional layers learn mid- to high-order features, such as edges, which are more specific to the dataset in the target domain than the more generic features from the dataset in the source domain. Because the parameters in the last convolutional layer are unfrozen and updated during backpropagation, FT typically requires more computational time than FE. The procedures for TL using FE and FT are shown in Figure 3. Similar studies using the TL approach for SD assessment include real-time crack detection using unmanned aerial vehicles [24], building defect detection [26], concrete bridge surface damage detection [27], and crack segmentation on masonry surfaces [28].

Well-established versions of VGGNet are VGG16 (16 layers) and VGG19 (19 layers), which contain 138 and 144 million parameters, respectively. The VGGNet architecture comprises five convolutional blocks, with each block containing two or more convolutional layers and a max-pooling layer. ReLU activation functions are provided in all hidden layers, and the output comprises three fully connected layers with softmax functions. Applications of pre-trained VGGNets through TL include crack detection [29], bolt-loosening detection [30], steel damage condition assessment [31], building defect detection [26], and post-earthquake SD assessment [6,7].

The inception network is engineered significantly for performance improvement and has a relatively lower error rate compared with VGGNet. Different versions of the inception modules that have evolved include V1, V2, V3, and V4. Within the inception block, parallel filter operations are applied to the input from the previous layer, followed by depth-wise concatenation of the filter outputs. Previous applications of inception networks in image classification include crack detection [32] and tunnel rock structure identification [33].

Xception is an extension of inceptionV3, where the convolutional layers are replaced with depth-wise separable convolutions. It comprises blocks of convolution and separable convolution followed by batch normalization and max-pooling layers. Use cases of Xception include aerial visual geolocalization [34] and construction site safety [35].

ResNet is a deep neural network that is based on residual learning. ResNet50 comprises 50 main layers and 177 layers, whereas ResNet101 comprises 101 main layers and a total of 347 layers. ResNet has been successfully applied to bridge component extraction [36] and road crack detection [37].

MobileNet comprises a class of efficient models based on depth-wise separable convolutions, which are widely used for mobile applications. The MobileNet block typically comprises batch normalization, 3 × 3 depth-wise convolution, 1 × 1 convolution layers, and ReLU activation. Because MobileNets have fewer parameters and a higher classification accuracy, they are typically adopted to build lightweight deep neural networks. MobileNet is used for road damage detection [38] and post-hurricane aerial damage assessment [39]. The pseudocode of the algorithm for the CNN model is presented in Table 3.

Each model was trained with an SGD optimizer on a high-performance computer with an Intel (R) Core i7-8700 CPU @ 3.20 GHz, 32 GB RAM, and an NVIDIA RTX Quadro 5000 GPU in a Keras/TensorFlow environment. A preliminary experiment was performed on the dataset based on a learning rate of 0.0001, a momentum set of 0.9, and a batch size of 32 images. The number of training epochs was set to 60 for all the experiments, and the images were resized to 224 × 224 × 3 before training. The validation set was used to tune the hyperparameters and optimize the weights of the CNN model. During FT, only the final convolutional block of the pre-trained model was retrained. In addition, a dropout rate of 0.5 was used between fully connected dense layers to reduce overfitting. To avoid overfitting problems, data augmentation techniques such as image cropping, standardization, random shifts, and horizontal image flips were implemented during model training. The properties of the pre-trained CNN models considered in this study are listed in Table 4.

## 4. Results and Discussion

Several experiments were performed to establish the performance of the 12 CNN models on image datasets. The potential of both FE and FT TL methods for structural image classification is analyzed in this section.

### 4.1. FE with Bottleneck Features

**Figure 4:**
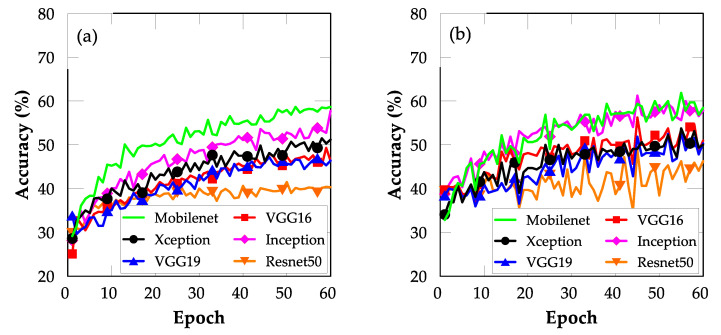
Plots of accuracy for models trained using FE TL: (**a**) training and (**b**) validation.

Figure 4a,b show the FE results of FE using the six pre-trained models. The pre-trained MobileNet CNN model exhibited training and validation accuracies of approximately 59% and 58.4%, respectively. Thus, it outperformed all the other models.

Notably, the ResNet50 model demonstrated categorically unsatisfactory performance compared with the other models, indicating that the architecture of the ResNet50 model was deeper and more difficult to train than those of the other models. Similarly, the VGG16 and VGG19 models demonstrated unsatisfactory performance, which might be due to their shallow architectures. However, the superior accuracy of MobileNet suggests that it is the best model for mobile application development.

### 4.2. FT

**Figure 5:**
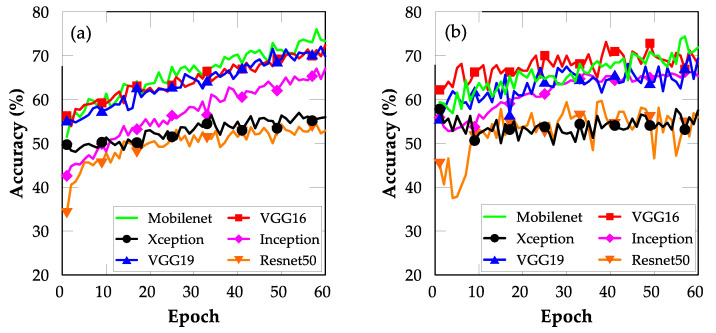
Plots of accuracy for models trained using FT TL: (**a**) training and (**b**) validation.

The FT results for the six pre-trained models are shown in Figure 5a,b. Similarly, the pre-trained MobileNet CNN model outperformed the other models in terms of its training and validation accuracies of approximately 73.4% and 71.8%, respectively.

### 4.3. Comparison between FE and FT

**Figure 6:**
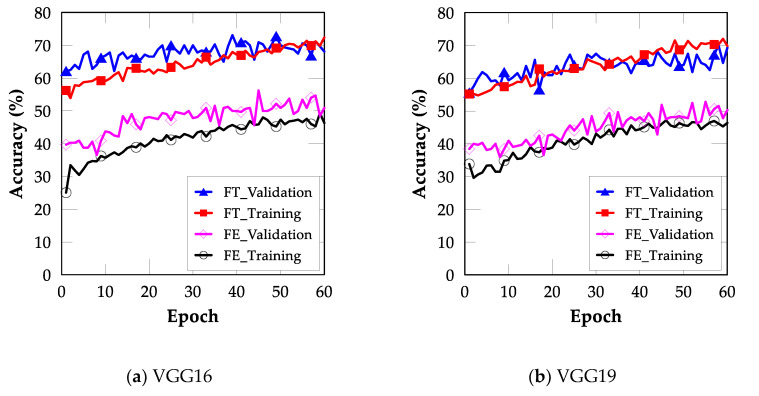

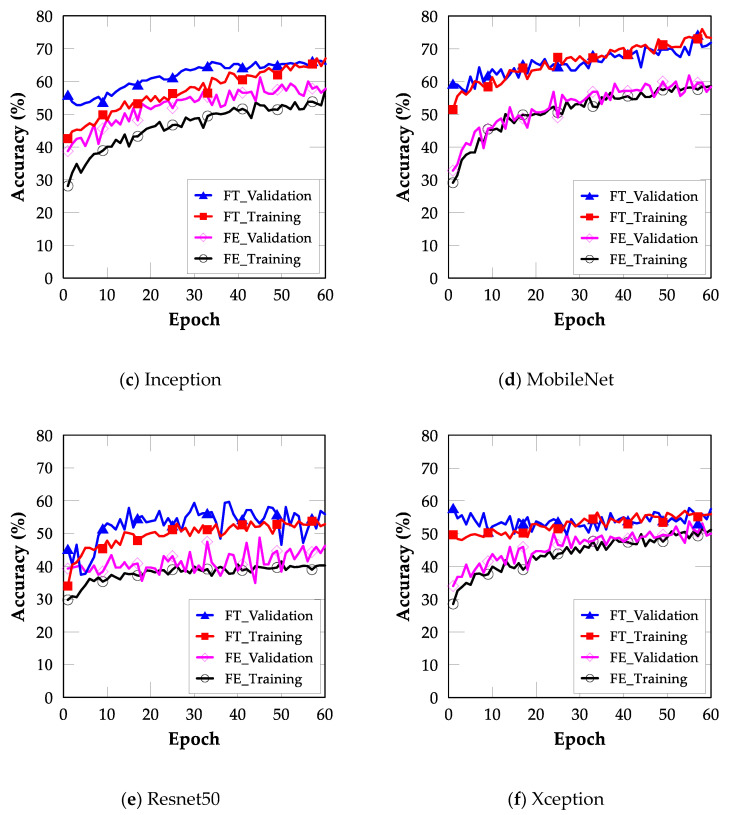
Training and validation accuracies of various CNN models implemented through TL: (**a**) VGG16, (**b**) VGG19, (**c**) Inception, (**d**) MobileNet, (**e**) ResNet50, and (**f**) Xception models.

The FT method performed better than the FE method for all models and datasets considered in this study. However, the FT method is computationally expensive because it involves retraining one convolutional block. Figure 6 shows the training and validation accuracies for each model implemented through TL.

**Figure 7:**
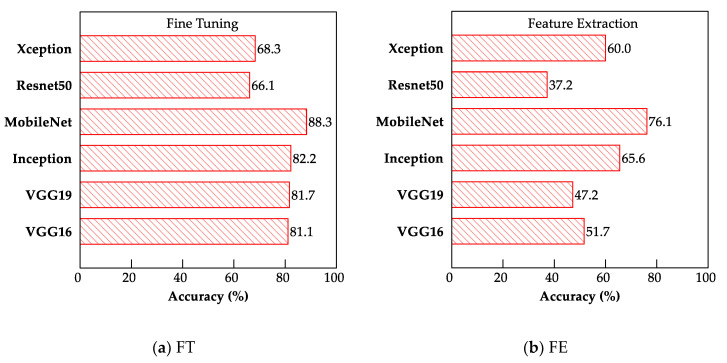
Bar charts showing testing accuracies for (**a**) FT and (**b**) FE of CNN models.

The results of the testing accuracy analyses for all the models are presented as bar charts in Figure 7.

### 4.4. Comparative Study: Effect of Dataset Size on Fine-Tuned Model

**Figure 8:**
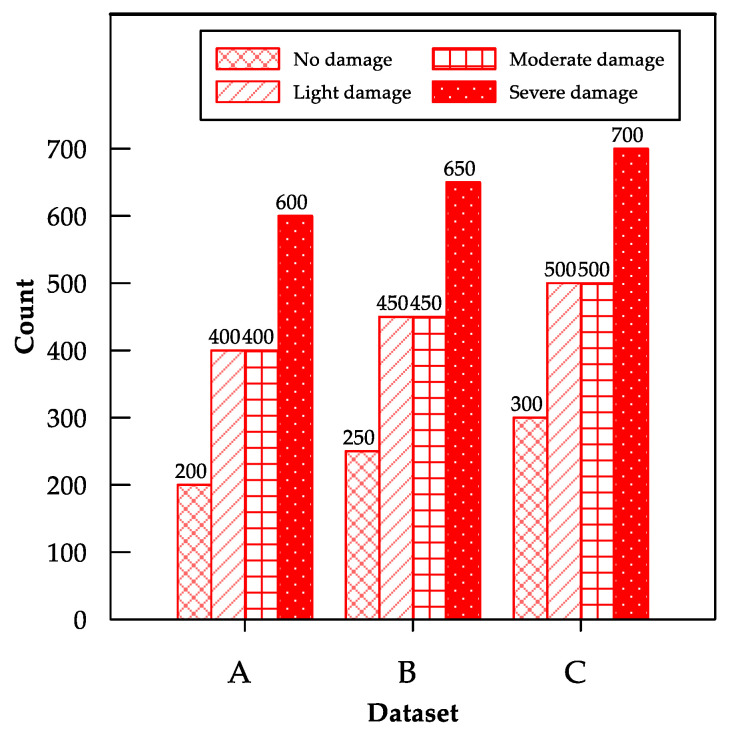
Summary of training and validation datasets for comparative study.

Because DL models are generally data intensive, the effect of data size on the performance of the fine-tuned MobileNet model was examined by gradually increasing the amount of training image data (Figure 8).

**Figure 9:**
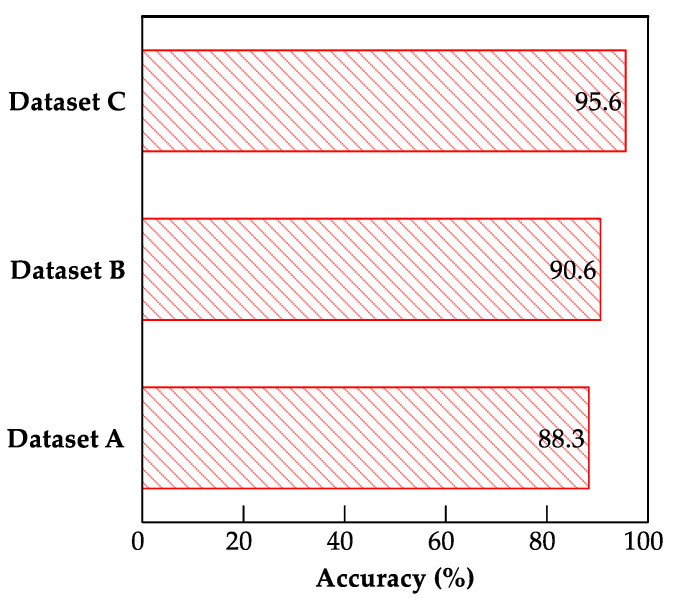
Bar charts showing testing accuracies for datasets A, B, and C using fine-tuned MobileNet CNN model.

An increase in the number of training images considerably affected the performance of the model (Figure 9). For example, the testing accuracies of the fine-tuned MobileNet model for datasets A, B, and C were 88.3%, 90.6%, and 95.6%, respectively. Thus, we infer that adding more training data to the model can improve its validation accuracy. Moreover, this is consistent with the findings of [6], which suggests that increasing the data and fine-tuning the convolutional blocks can improve the model performance.

**Figure 10:**
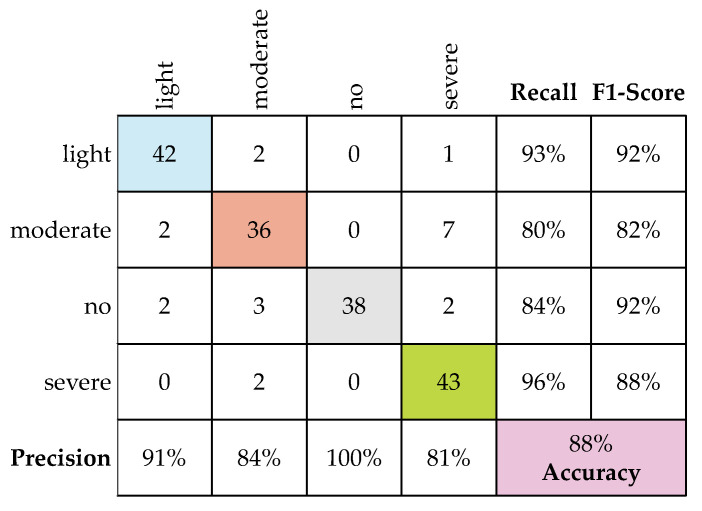
Confusion matrix for the fine-tuned MobileNet CNN model.

The fine-tuned MobileNet CNN model, which exhibited optimal performance with a testing accuracy of 88.3%, was selected for deployment in a web-based application for earthquake-damage classification. Figure 10 shows plots of the confusion matrix used to evaluate the model performance of the testing images.

To assess the performance of the fine-tuned MobileNet CNN model, the testing accuracy was compared with those of various CNN architectures used for similar SD classification tasks. A comparison of the different models with the optimal model is presented in Table 5. Accuracy can be expressed as the ratio of the true predictions to the total predicted cases in the dataset. The precision metric measures the classifier’s ability to correctly identify positive classes. The recall metric is the ratio of positive instances that are correctly detected by the classifier to the total number of positive instances. The mathematical expressions for accuracy, precision, recall, and F1 score are shown in Equations (1a)–(1d), respectively.

(1a) $$Accuracy\;=\;\frac{TP\;+\;TN}{TP\;+\;TN\;+\;FP\;+\;FN}$$

(1b) $$Precision\;=\;\frac{TP}{TP\;+\;FP}$$

(1c) $$Recall\;=\;\frac{TP}{TP\;+\;FN}$$

(1d) $$F_{1}\;=\;2\;\times \;\frac{precision\;\times \;recall}{precision\;+\;recall}\;=\;\frac{TP}{TP\;+\;\frac{FN\;+\;FP}{2}}$$

where TP = number of true positives, TN = number of true negatives, FP = number of false positives, and FN = number of false negatives.

The proposed model was trained on datasets containing images of all structural members similar to those used by Gao and Mosalam [6], which involve extremely noisy backgrounds. However, the dataset considered by Pan and Yang [8] contained only images of reinforced concrete structural columns with less background noise; hence, their approach afforded higher accuracy.

**Figure 11:**
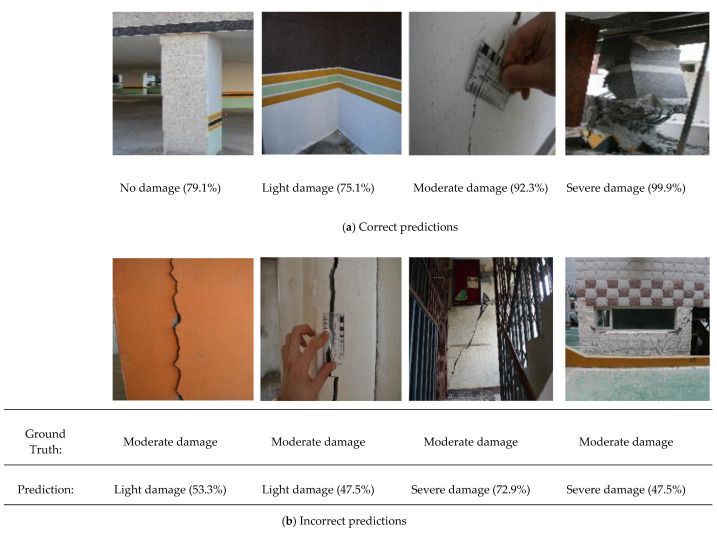
Sample testing images of structural damage with predicted probability for cases of (**a**) correct and (**b**) incorrect predictions.

A sample of the testing images with predictions obtained from the fine-tuned MobileNet model is shown in Figure 11. Despite the varying inclination of the camera view and light intensity of the images, the model successfully predicted the SD classes, with extremely few instances of incorrect predictions. For example, it predicted light damage in two cases, as shown in Figure 9b, instead of the ground truth, which indicates moderate damage. This misclassification can be attributed to the overlapping of hairline cracks (light damage) and wide cracks (moderate damage) in the images. Similarly, moderate damage was occasionally misclassified as severe damage, which might be attributed to background noise, such as the presence of iron bars and large window voids in the images. Hence, a more robust bounding box object-detection technique or other forms of damage localization in the model should be considered to overcome this deficiency.

The accuracy of computer-vision-based SD assessment is mainly affected by the complexity of the structure and damage. The damage assessment results can be affected by the varying lighting conditions, occlusion, and insufficient known reference points on a damaged structure that can be used for comparison with pre-damage images to accurately assess damage levels. Moreover, SD caused by debris and rubble can often be difficult or impossible to detect using computer-vision algorithms alone.

### 4.5. Visualization and Localization of Damage Using Grad-CAM

Grad-CAM is a visualization technique that visualizes and clarifies predictions from large classes of CNNs to render them more transparent. Initially published by Selvaraju et al. [40], Grad-CAM uses the gradient of the target concept in the last convolution layer to create an approximate localization map that highlights the areas of interest to predict the concept.

**Figure 12:**
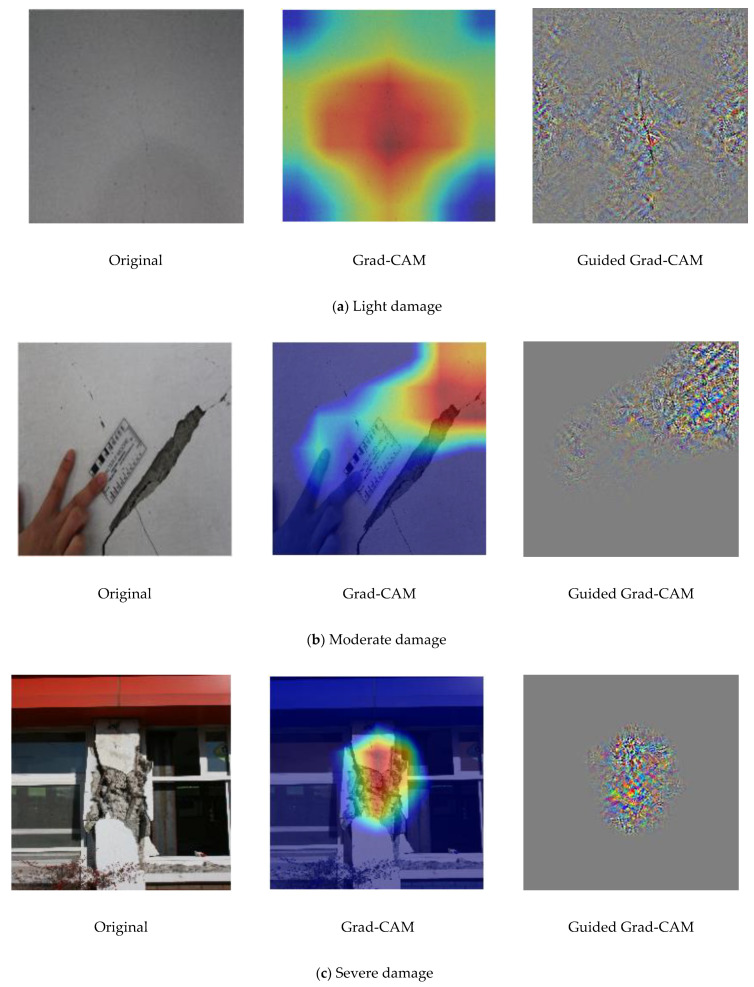
Representative images illustrating damage visualization and localization analyses via gradient-weighted class activation mapping (Grad-CAM) methods for images of (**a)** light, (**b**) moderate, and (**c**) severe damage.

Grad-CAM was used to extract gradients from the fine-tuned MobileNet CNN model in the final convolutional layer to generate localization maps that identify relevant regions in the test images. This visualization technique is advantageous over the conventional bounding-box method, which is subjective as it requires manual annotations. The heat maps generated via Grad-CAM exhibit smooth boundaries, which provide insight into the precise location of defects or damage in the SD images. Figure 12 shows representative images from different SD classes localized using the Grad-CAM and guided Grad-CAM methods.

The mislocalization of the moderate damage image (in Figure 12b) is attributed to the lower predicted probability (88.83%) for this image compared with the light (98.13 %) and severe (97.29 %) damage images.

### 4.6. Damage Severity Measurement

Following the approach of Li et al. [16], damage severity was quantified by assigning a damage assessment value (DAV) obtained from Grad-CAM-based damage detection map (DDM). Mathematically, for an input image *x* with output damage class *y_D_* of the VGG19 CNN model, the gradient-based weight parameter *w_k_* is the aggregate of gradients in *y* with respect to *f^k^_(i,j)_* for *i* and *j,* and is expressed as follows:

(2) $$w_{k}\;=\;\frac{1}{14\;\times \;14}\;\underset{i,j}{\sum }\frac{\partial y_{D}}{\partial f_{\left(i,j\right)}^{k}},$$

where *f^k^_(i,j)_* is the *k-th* feature map in the last convolutional layer (which measures 14 × 14 × 512 in this study), *i* = 1, …, 14, *j* = 1, …, 14, and *k* = 1, …, 512.

For feature maps *f^k^* and the corresponding weights *w_k_*, a 14 × 14 matrix S can be defined as

(3) $$s_{i,j}\;=\;ReLU(\underset{k}{\sum }w_{k}f_{\left(i,j\right)}^{k}),$$

where ReLU() eliminates the effects of negative values and emphasizes positive values.

In the DDM, numerical values are assigned to quantify the damage severity based on the pixel intensity. Higher pixel intensities reflect more severe damage and are represented by a heat map in the DDM. The average numerical values obtained from the heat map of an image are regarded as the overall DAV, which quantifies the damage severity of the image. Hence, a high DAV indicates severe damage and is defined as follows:

(4) $$\mathrm{DAV}\;=\;\frac{1}{14\;\times \;14}\;\underset{k}{\sum }s_{i,j},$$

where *s_i,j_* represents the elements in matrix S, and the dimensions of S are 14 × 14.

The DAV ranges between 0 (no damage) and 1 (total collapse).

**Figure 13:**
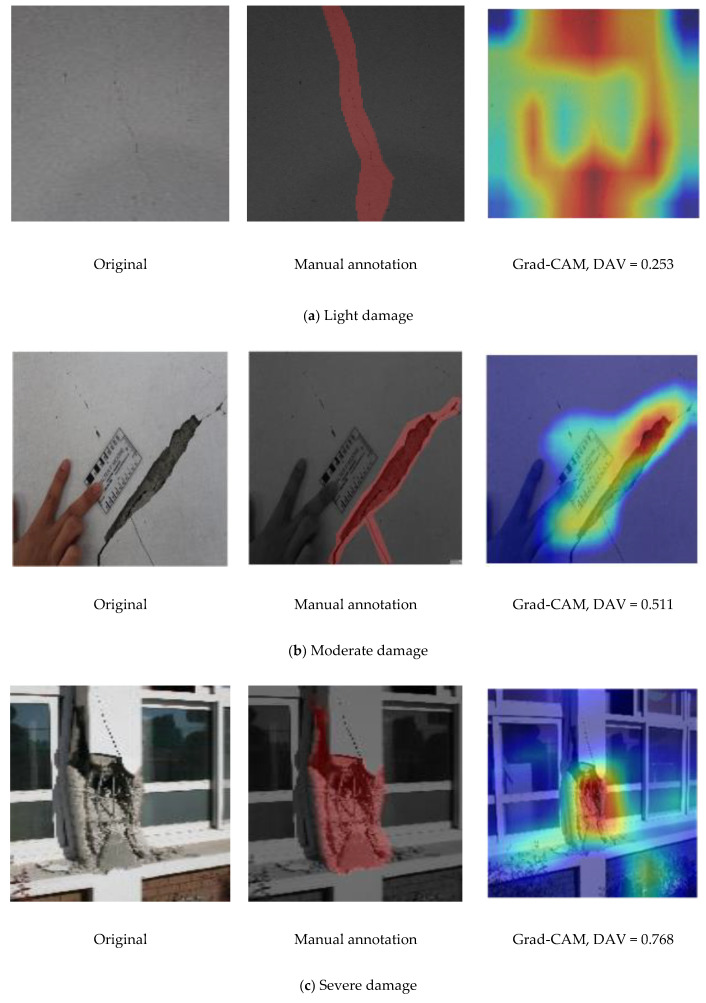
Sample images with annotations for severity and corresponding damage assessment value (DAV) scores for images of (**a**) light damage, (**b**) moderate damage, and (**c**) severe damage.

An annotation tool, known as LabelMe [41], accessible at http://labelme.csail.mit.edu, is used to annotate the SD images manually. The numerical values are assigned as follows: no damage = 0, light damage = 0.25, moderate damage = 0.5, severe damage = 0.75, and total collapse = 1 [42]. The annotated sample images are shown in Figure 13, along with their corresponding severity values.

## 5. Development of CNN Model as Interactive Web Application

**Figure 14:**
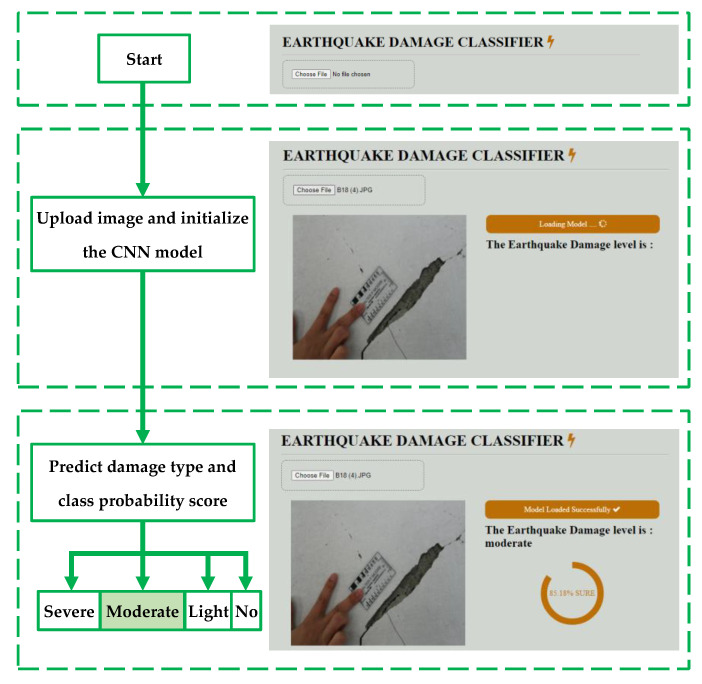
Graphical user interface for web-based application that integrates optimal MobileNet damage classifier model.

Access to trained DL/machine learning models in portable and interactive formats can facilitate real-time practical damage assessments. As shown in Figure 14, the optimal earthquake damage classifier model is converted to a Tensorflow.js compatible format and deployed as a web application with an easy-to-use graphic user interface. Tensorflow.js, built on the Tensorflow framework, facilitates the conversion of machine learning models to JavaScript formats, accessible through web browsers [43]. In addition to the superior accuracy of the optimal MobileNet CNN model, its lightweight size renders it the best model among all the trained models. An interactive web application is a useful tool that allows users to upload SD images and rapidly determine the class of SD with the corresponding confidence level of prediction. The prediction probability is computed based on the softmax function, as shown in Equation (5).

(5) $$P(y_{i})\;=\;\frac{exp\left(y_{i}\right)}{\sum_{j\;=\;1}^{n}\exp\left(y_{j}\right)},$$

where *P*($y_{i})$ is the prediction probability of class *i*, $y_{i}$ is the output score for class *i*, and *n* is the number of classes.

The trained CNN model is accessible to web browsers at https://bit.ly/3hXRyyc. This allows emergency responders to rapidly assess post-earthquake damage and make informed decisions regarding resource allocation. In addition, users can upload images captured at ground level from different sources to identify the SD and further validate the performance of the proposed earthquake-damage-classifier model.

## 6. Conclusions and Recommendations

Artificial intelligence for post-earthquake inspections and reconnaissance has recently received significant attention, owing to its exponential increase in computational capabilities and the inherent potential of artificial intelligence to address the disadvantages associated with manual inspections, including subjectivity. In this study, we used data from the 2017 Pohang earthquake to demonstrate the potential of automated DL for rapid and accurate inspections of post-earthquake damage with insignificant human input.

Our key findings are as follows:

1. The FT method outperformed the FE method for all the CNN models evaluated. However, the FT method is more computationally complex than the FE method because it involves retraining one convolutional block.
2. The MobileNet model exhibited the best performance for both the FE and FT TL methods, exhibiting testing accuracies of 76.1% and 88.3%, respectively. The superiority of the MobileNet model in performing classification promoted its deployment as a web-based application for earthquake-damage classification.
3. The web application successfully predicted the damage class in new images of seismic damage with high certainty. In addition, interactive web pages can rapidly and automatically classify SD from earthquakes, thereby facilitating decision making in response to earthquakes.

In this study, we demonstrated the potential of automated DL to facilitate post-earthquake damage inspections and surveys. Despite the limitations of this study, including the lack of a large and sophisticated training dataset and the complexity of the four damage classes, future studies will be conducted that focus on establishing a large benchmark dataset with high-quality annotations, such as the PEER Hub ImageNet [12]. In addition, future experiments, involving unmanned aerial vehicles, will be performed to capture real-time images from SD sites that can be sent to a webpage interface for fully automated damage assessment.

## Figures and Tables

**Table 1:** Categorized summary of the image dataset.

Image Source	No Damage	Light Damage	Moderate Damage	Severe Damage
Pohang (2017) [4]	49	294	187	551
Haiti (2010) [1]	52	55	174	127
Nepal (2015) [3]	152	153	123	255
Taiwan (2016) [2]	3	99	27	34
Ecuador (2016) [5]	4	108	115	188
Total	260	709	626	1155

**Table 2:** Categorized summary of images in training, validation, and testing datasets.

Image	No Damage	Light Damage	Moderate Damage	Severe Damage
Training	160	320	320	480
Validation	40	80	80	120
Testing	45	45	45	45
Total	245	445	445	645

**Table 3:** CNN model algorithm pseudocode.

CNN Algorithm
Programming language used for implementation: Python. Libraries for CNN model building: Tensorflow and Keras. Libraries used for image augmentation: OpenCV and computer vision library. Libraries used for visualizations: Matplotlib and 2D graph tool. 1. Let ***X*** be the input image of the batch and **y** be the label for the image ***X***. 2. Extract features from the image using a CNN algorithm. Freeze all the pretrained convolutional blocks to serve as a feature extractor or fine tune by unfreezing the last convolutional blocks. Obtain feature maps of the first layer ***a*_0_** after passing the image into the convolution layer with 7 × 7 filters and apply batch normalization function along with ReLU function. Apply the global average pooling function to the output tensor ***a*_0_**. Flatten the output to obtain a feature vector. 3. Execute the feature classification network on the feature vector. Initialize the weight ***w*** and bias ***b*** arrays of the linear network comprising 256 neural nodes. Add 50% dropout to serve as a regularizer and reduce overfitting. Perform ***z*** = ***w***. ***a****_feature_* + ***b***. Perform ReLU activation function ***a^l^*** = **max** (***z***, **0**). Initialize weight ***w*** and bias ***b*** arrays of linear network with four neural nodes. Perform ***z*** = ***w***. ***a^l^*** + ***b***. Perform ReLU activation function ***a^l^*** = **max** (***z***, **0**). Apply softmax function on ***a^l^*** to obtain the probability distribution of the four classes: no, light, moderate, and severe damage.

**Table 4:** Comparison of proposed pre-trained CNN models.

Model	No. of Parameters	Depth of Layers	Size (MB)
VGG16	138.4 M	16	528
VGG19	143.7 M	19	549
Inception	23.9 M	189	92
Xception	22.9 M	81	88
ResNet	25.6 M	107	98
MobileNet	4.3 M	55	16

**Table 5:** CNN-based SD classification models compared with current study.

Task Description	Algorithm	Accuracy (%)	* Precision (%)	* Recall (%)	References
Classification of damage in all structural members	VGG16	68.8	-	-	[6]
Classification of damage in columns only	ResNet50	87.47	-	-	[8]
Classification of damage in all structural members	MobileNet	88.3	89	88.2	Current work

* Values are not provided in the referenced study.

## Abbreviations

The abbreviations used in this manuscript are as follows:

CNN	Convolutional neural network
DAV	Damage assessment value
DDM	Damage detection map
DL	Deep learning
FE	Feature extraction
FT	Fine-tuning
GPU	Graphic processing unit
Grad-CAM	Gradient-weighted class activation mapping
OLeNet	Optimized LeNet
PEER	Pacific Earthquake Engineering Research
ReLU	Rectified linear unit
SD	Structural damage
TL	Transfer learning
VGG	Visual geometry group
